# Potential Effects of Chrysin on MDA-MB-231 Cells

**DOI:** 10.3390/ijms11031057

**Published:** 2010-03-11

**Authors:** Teh Ban Hong, Anizah Rahumatullah, Thaneswary Yogarajah, Maimunah Ahmad, Khoo Boon Yin

**Affiliations:** 1 Institute for Research in Molecular Medicine (INFORMM), Universiti Sains Malaysia, 11800 USM, Penang, Malaysia; E-Mails: banhong86@hotmail.com (T.B.H.); anirah_arshypop82@yahoo.com (A.R.); ythanes13@yahoo.com (T.Y.); mai_ahmad@notes.usm.my (M.A.); 2 School of Biological Sciences, Universiti Sains Malaysia, 11800 USM, Penang, Malaysia;

**Keywords:** chrysin, cell growth inhibition, lipid accumulation, apoptosis, PPAR mRNA expression, ER-negative breast cancer

## Abstract

This study aims to elucidate the effects of chrysin on human ER-negative breast cancer cell line, MDA-MB-231. The study demonstrated that treatment of MDA-MB-231 cells with 20 μM chysin for 48 h significantly inhibited the growth of MDA-MB-231 cells and induced cytoplasmic lipid accumulation in the cells, but that the observed of cell death was not caused by apoptosis. The expression of PPARalpha mRNA in chrysin-treated MDA-MB-231 cells was significantly increased, which was likely associated to the proliferation of the cells post chrysin treatment.

## 1. Introduction

**Figure 1.**
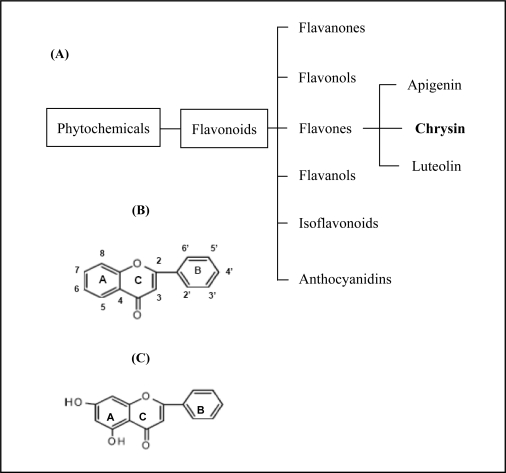
(**A**) Chrysin is in the flavones group of the flavonoids. (**B**) The generic structure of flavone type of flavonoids. The structure comprises of ring A, ring B and ring C. (**C**) Chrysin has hydroxyl group at position 5, 7 in ring A [4].

Chrysin is a natural phytochemical flavonoid belonging to the flavone group (Figure 1) which is ubiquitously found in fruits and vegetables [1,2]. Besides giving flavour and colour, flavonoids, including chrysin, have recently been identified as displaying important biological roles in nitrogen fixation and chemical defence. Chrysin, in particular, has been shown to have a broad range of pharmacological effects, including anti-oxidation, anti-viral and anti-inflammatory properties in a diverse range of human and rat cells [3,4]. Chrysin has also been observed to reduce the risk of cancer and other major chronic diseases [1], and has been observed to display anti-cancer properties on breast cancer cells, though the mechanisms have not been well established [5]. To further elucidate these mechanisms, our study aimed to evaluate the effects of chrysin treatment on human estrogen receptor (ER)-negative breast cancer cells using MDA-MB-231 cell line as a model.

MDA-MB-231 is an ER-negative breast cancer cell line that was derived from a metastatic adenocarcinoma on the mammary glad of a 51-year-old Caucasian woman, according to the ATCC. The cells are highly aggressive, invasive and poorly-differentiated [6]. It is the most commonly used cell line for human ER-negative breast cancer studies. The ER-negative breast cancer is specifically studied due to a lack of cellular targets compared with ER-positive breast cancer, which can be effectively inhibited by targeting the estrogen receptor with anti-estrogen agents such as tamoxifen. The proliferation of ER-negative breast cancer cells is not affected by estrogen, which negates the use of anti-estrogen therapies. This makes the search for potential anti-cancer agents to treat ER-negative breast cancer all the more urgent and important.

In this study, the viability of MDA-MB-231 cells treated with different concentrations of chrysin and for different treatment times was evaluated using trypan blue assay. Determination of the preliminary viability effect of chrysin, in this study, was sourced from previous studies described in Table 1 [5,7–10]. Once the effective concentration and treatment time of chrysin were determined, several tests were performed to discern the state of cell differentiation and the mechanisms of cell death. The cell differentiation in chrysin-treated MDA-MB-231 cells was performed using Oil-red O staining, whereas the effect of chrysin on apoptosis was examined using DNA fragmentation and TUNEL assays. Finally, the expression of peroxisome proliferator-activated receptor (PPAR) alpha, delta and gamma mRNAs was semi-quantified using RT-PCR to identify the levels of PPAR mRNAs, which may potentially be affected by the chrysin treatment. This study seeks to elucidate the anti-cancer effects and cellular mechanisms of chrysin in order to support a rational use on breast cancer treatment. The data obtained from this study may provide valuable information to improve treatment for aggressive, invasive and poorly-differentiated human ER-negative breast cancer.

## 2. Results and Discussion

**Figure 2.**
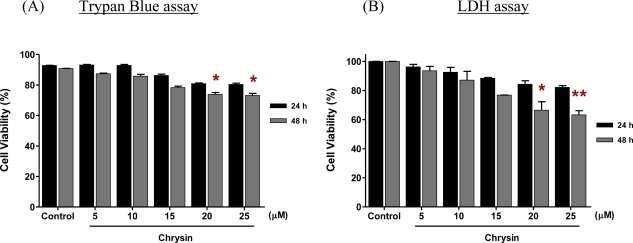
The effect of chrysin on the growth of MDA-MB-231 cells for 24 h and 48 h assessed using (**A**) the trypan blue assay and (**B**) the LDH assay. The data are mean ± SD of triplicate cultures. Analysis of one-way ANOVA was used to compare the viability of MDA-MB-231 cells in different concentrations of chrysin compared to control at each treatment time. *p* < 0.05 was regarded as statistically significant.

Figure 2A shows the viability of MDA-MB-231 cells treated with different concentrations of chrysin for 24 h and 48 h. Chrysin was observed to inhibit the growth of MDA-MB-231 cells up to 48 h treatment in a dose-dependent manner. After 24 h treatment, the percentage of cell viability ranged from 92.8% to 80.9% when the MDA-MB-231 cells were treated with the lowest (5 μM) to the highest (25 μM) concentration of chrysin, respectively. However, the reduction of cell viability at all concentrations at this treatment time was not statistically significant. The growth of MDA-MB-231 cells was significantly inhibited when the cells were treated with 20 μM of chrysin for 48 h, where the cell viability was reduced to 73.9% (*p* < 0.05). Chrysin at 25 μM concentration exhibited a similar significant inhibitory effect upon the 20 μM concentration with a reduction of cell viability to 73.1% (*p* < 0.05). Thus, 20 μM chrysin for 48 h was used as the effective concentration and treatment time for subsequent cell differentiation and cell death experiments.

Trypan blue, use in this study, is a fast and reliable colorimetric method to assess the cell viability. The assay, in which trypan blue is excluded from live cells but accumulated in dead cells, is one of the most commonly used methods for calculating the percentages of live and dead cells in a given cell population [16]. The trypan blue assay is also useful in DNA content analysis, which cannot be analysed by some other methods, such as lactate dehydrogenase (LDH) release assay and assay determine the level of tetrazolium salt (MTT). In this study, the LDH assay was performed to measure the growth inhibitory effect of chrysin on MDA-MB-231 cells after 48 h treatment in order to validate the original results of the trypan blue assay. The results of these two assays were almost identical (Figure 2B).

**Figure 3.**
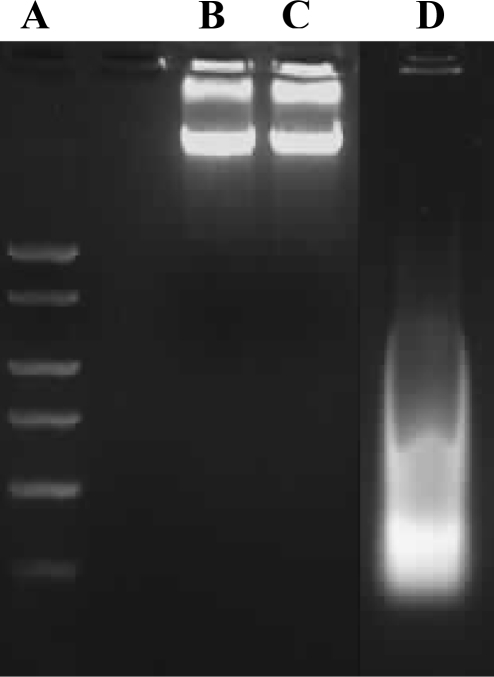
Fragmentations of the genomic DNA of MDA-MB-231 cells post treatments. (**A**) DNA marker. The cells were treated with (**B**) 0.1% DMSO or (**C**) 20 μM chrysin for 48 h. (**D**) positive control provided by the kit. Fragmented DNA was extracted and analysed using 1% agarose gel.

**Figure 4.**
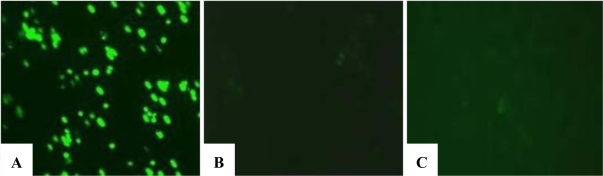
TUNEL staining of MDA-MB-231 cells post treatments. (**A**) The MDA-MB-231 cells, treated with DNase I recombinant, showed positive staining of apoptotic nuclei. The MDA-MB-231 cells, treated with (**B**) 0.1% DMSO or (**C**) 20 μM chrysin for 48 h, did not show the positive staining of apoptotic nuclei.

**Figure 5.**
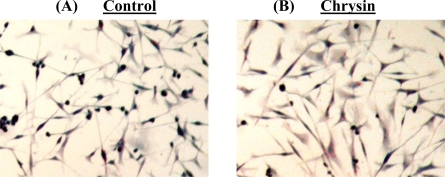
Cytoplasmic lipid accumulation post treatments. The MDA-MB-231 cells, treated with (**A**) 0.1% DMSO or (**B**) 20 μM chrysin for 48 h, showed cytoplasmic lipid accumulation in the cells.

Cell death, particularly apoptotic DNA fragmentation was examined in chrysin-treated MDA-MB-231 cells because the reduction of cell viability was likely to be an outcome of mitochondrial dysfunction, which would eventually lead to the cell death, according to Mosmann [11]. However, in our study, the cell death experiments indicated that the growth of chrysin-treated MDA-MB-231 cells was not inhibited through apoptosis. The apoptotic DNA fragmentation test and fluorescence microscopy using TUNEL assay showed no apoptotic cells were detected in the chrysin-treated MDA-MB-231 cells (Figures 3 and 4), though cytoplasmic lipid accumulation was observed in the chrysin-treated MDA-MB-231 cells (Figure 5). The DNA fragmentation assay showed no cleavage of chromosomal DNA into oligonucleosomal size fragments and the TUNEL staining showed no green DNA break staining, both of which are positive indicators of apoptosis. The high rate of lipid accumulation in the cytoplasm of chrysin-treated MDA-MB-231 cells observed through the Oil-Red O staining in Figure 5 indicates that the cells may undergo cell differentiation which may be due to the increased expression of PPARs (lipid sensors) in the cytoplasm after chrysin treatment [14]. However, the lack of effect of chrysin on apoptosis in MDA-MB-231 cells is not known. Thus, the expression of PPAR mRNAs was subsequently determined in chrysin-treated MDA-MB-231 cells using semi-quantitative RT-PCR. Only mRNA expression was determined in this study because we would like to highlight the initial changes in the mRNA expression level which is an important indicators of the early events in cell regulation.

**Figure 6.**
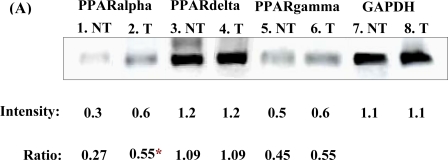

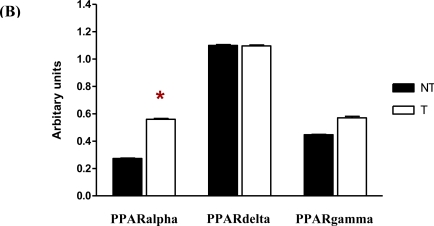
(**A**) The PCR bands of PPARs and GAPDH in control DMSO-treated (NT) and chrysin-treated (T) MDA-MB-231 cells at 48 h treatment. The GAPDH gene, which was used as internal control, indicates an equal loading of cDNA samples. The results shown are representative of three independent experiments. (**B**) The densitometric values of the bands are shown as mean±SD from three independent experiments. The student’s t-test was used to compare the intensity of the PPARs PCR bands between (T) and (NT). *p* < 0.05 was regarded as statistically significant.

Following semi-quantitative RT-PCR, the gels showed that single bands were detected for the PPARalpha, PPARdelta, PPARgamma and GAPDH bands in both the chrysin-treated and control DMSO-treated MDA-MB-231 cells (Figure 6A). The PCR reactions resulted in bands of 344 bp for PPARalpha (Lane 1–2), 566 bp for PPARdelta (Lane 3–4), 228 bp for PPARgamma (Lane 5–6) and 137- bp for GAPDH (Lane 7–8). In Figure 6B, treatment of MDA-MB-231 cells with 20 μM chrysin for 48 h showed a significant increase of the PPARalpha mRNA (*p* < 0.05), as reflected in the intensity of the band compared with the control samples (ratio intensity of PPARalpha and control samples were 0.55 and 0.27, respectively). There was also an increase in the intensity of the PPARgamma band in chrysin-treated MDA-MB-231 cells but it was not statistically significant when compared with the control samples (ratio intensity of PPARgamma and control samples were 0.55 and 0.45, respectively). This phenomenon indicates that increased expression of PPARalpha mRNA may directly or indirectly plays an important role in MDA-MB-231 cells when the cells were inhibited by chrysin treatment.

Our study demonstrated that chrysin inhibited the growth of MDA-MB-231 cells in a dose-dependent manner. The inhibition of cell growth was consistent with that reported by Parajuli *et al*. [5], who used chrysin extracted from the leaf, stem and roots of various *Scutellaria* plants. According to their study, chrysin at a concentration of 100 μM significantly inhibited the growth of MDA-MB-231 cells after 4 days’ treatment (about 50.0%, *p* < 0.05). In our study, 20 μM chrysin, at 97% purity, was sufficient to inhibit the growth of MDA-MB-231 cells after two days’ treatment (73.9%, *p* < 0.05). A 48 h treatment of 20 μM chrysin was used to determine the preliminary mechanisms of the chrysin on MDA-MB-231 cells, because this was the condition under which the induction of early growth inhibition in MDA-MB-231 cells was statistically significant (Figure 2). Increase of chrysin concentration did not increase the growth inhibition but rather triggered the accumulation of the drug crystal in the culture. Moreover, the treatment time used in this preliminary study was no longer than 48 h because only these time points will be used to determine the effects of chrysin and ciglitazone treatment in combination on MDA-MB-231 cells in the follow-up study, in which a 48 h treatment of 20 μM ciglitazone induced about 50% growth inhibition on MDA-MB-231 cells, in contrast to its growth inhibition of about 60% in normal breast cells. When chrysin and ciglitazone were used in combination, ciglitazone was needed in a lower dose, and less cytotoxicity on normal breast cells was observed after 48 h treatment (data not shown).

As 48 h is sufficient to assess the effect of chrysin on MDA-MB-231 cells, a longer treatment time was not necessary for our preliminary optimisation study. Indeed, study of Parajuli *et al*. (2009) demonstrated that chrysin at a concentration of 100 μM significantly inhibited the proliferation of MDA-MB-231 cells after 4 days treatment, but no apoptosis was reported in the study when the mechanism was assessed by flow cytometry, indicating that the increase of chrysin concentration and treatment time did not augment its effects on MDA-MB-231 cells. Moreover, although IC50 is the most common representative index of the dose-response curve to assess the half maximal effective concentration of a drug, some other endpoints, such as IC20 and IC80, are also necessary to assess the early inhibitory effect of a drug after certain specified exposure time.

Our study also revealed that the inhibition of cell growth was likely influenced by increased expression of PPARalpha mRNA in the cells. Although the level of PPARgamma mRNA was not significantly higher in the chrysin-treated MDA-MB-231 cells, its high expression in cytoplasm was likely directly or indirectly associated with increased mRNA level of PPARalpha in the treated cells. This is similar as demonstrated by Bocca [12] that PPARgamma-mediated pathway is not apparently involved on the growth inhibitory activity, but it could be involved *via* establishing the connection with PPARalpha pathway. Moreover, the expression of PPARalpha was recently demonstrated to be associated with tumorigenesis and its mRNA is detected expressing in MCF-7 and MDA-MB-231 cell lines [13]. The PPARalpha is predicted to regulate the cancer cell proliferation, though the role of PPARalpha in cellular apoptosis was not demonstrated and being studied as wide as the PPARgamma. In Suchanek *et al*. study [13], the expression of PPARalpha mRNA was regulated in MDA-MB-231 cells and the PPARalpha activation significantly increased the proliferation of the cells. The promotion of cell proliferation in MDA-MB-231 and MCF-7 following the PPARalpha activation was likely in stark contrast to the effects of PPARgamma-activating ligands that decreased the proliferation in these cells, according to the study. Indeed, the PPARalpha and PPARgamma activation had very different effects on the breast cancer cells. Therefore, further studies are required to determine the expression of PPARalpha and PPARgamma mRNAs which were likely induced when the growth of MDA-MB-231 cells was inhibited by the chrysin treatment. More data is needed to further reveal the mechanism of cancer cell growth inhibition mediated by the PPARalpha and PPARgamma as these transcription factors may play important roles as mediators when the cancer cell growth is inhibited by potential anti-cancer agents such as chrysin. Perhaps, the involvement of PPARalpha and PPARgamma can be related to AA signalling pathway which leading to an increase in cellular ceramide concentration [15]. Study of the interaction of these pathways could provide useful information to develop drug combinations that maximise the growth inhibitory effect on ER-negative breast cancer cells.

## 3. Experimental Section

### 3.1. Cell Culture

The MDA-MB-231 cell line was a kind gift from the John Hopkins Research Center. The cells were maintained with DMEM (*Gibco BRL*) supplemented with 10% (v/v) FBS (Gibco BRL), 100 U/mL penicillin and 100 mg/mL streptomycin (*Gibco BRL*). A MycoKill (1%) (PAA Laboratories Inc.) was added into the growth medium to prevent mycoplasma contamination. The growth medium was changed every 2–3 days and the cells were incubated at 37 °C in a humidified atmosphere of 5% (v/v) CO_2_. Chrysin (97% purity, Sigma-Aldrich) was dissolved with DMSO (Sigma-Aldrich) as 10^−2^ M stock. The drug stocks were then stored at −20 °C and further diluted to the working concentration with assay medium [DMEM supplemented with only 2% (v/v) FBS and antibiotics].

### 3.2. Determination of Effective Concentration and Treatment Time

The effect of chrysin on the growth of MDA-MB-231 cells was determined using the trypan blue assay. For this purpose, the MDA-MB-231 cells were seeded at a density of 1.0 × 10^3^ cells/mL in two 24-well cell culture plates (*NUNC*). When the cells of each well reached approximately 70% confluency, increasing concentrations of chrysin (5–25 μM) were added and the cells were incubated for 24 h and 48 h. DMSO (0.1%) was used as diluent control in this study. All control and experimental samples were cultured in triplicate. After 24 h treatment, the cells were harvested using trypsin/EDTA solution (Gibco BRL). The harvested cell suspension (0.9 mL) from each well was added with 0.1 mL of Trypan Blue Solution (Sigma-Aldrich) and was incubated at room temperature for 5 min. Living and dead cells were then counted using a hemocytometer and the cell viability (%) was calculated as below:

(1) Cell Viability (%) = (number of unstained cells per quadrant/total cells per quadrant)×100

Living cells were observed excluding the dye (unstained cells) whereas dead cells took up the blue dye. The same method was used to calculate the viability of MDA-MB-231 cells after 48 h of chrysin treatment. Analysis of one-way ANOVA was used to compare the viability of MDA-MB-231 cells in different concentrations of chrysin treatment with control DMSO for 24 h and 48 h. The lowest concentration at the effective treatment time which showed a significant reduction of cell viability was used as the effective concentration for subsequent experiments.

### 3.3. Validation of Trypan Blue Assay with LDH Assay

The trypan blue assay was validated using LDH assay where the cells were seeded at a density of 1.0 × 10^3^ cells/well in 24-well cell culture plates (*NUNC*). When the cells reached approximately 70% confluent, the cells were treated with increasing concentrations of chrysin for 48 h. The culture supernatants were then withdrawn for measurement of the LDH release using Roche Cytotoxicity Detection kit. The amount of LDH release in the culture supernatant was measured based on absorbance of color generated from each sample, according to the manufacturer’s instruction. The absorbance was read at 492 nm against 620 nm wavelength using ELISA reader (Thermo Labsystems). All treatments and control samples were analyzed in triplicate. The percentage of cell viability was calculated as (Sample value-Low control)/ (High control-Low control), where low control is the absorbance of cells cultured in assay medium and high control is the absorbance of cell cultured in the presence of 1% (v/v) Triton-X. The high control was then referred as sample with 100% LDH release.

### 3.4. Treatment of MDA-MB-231 Cells with Chrysin

After obtaining the effective concentration and treatment time for chrysin on the growth of MDA-MB-231 cells, 2 groups of cell cultures (control DMSO-treated and chrysin-treated) were prepared in triplicate for subsequent experiments. A total of 1×10^4^ cells/mL were seeded into 10 cm^2^ culture dishes for detection of apoptotic DNA fragments and extraction of total RNA for semi-quantitative RT-PCR. For detection of DNA fragmentation by fluorescence microscopy (TUNEL assay) and examination of cytoplasmic lipid accumulation, 1×10^3^ cells/well were seeded into 4-well culture slides. The cells were grown in growth medium until they were approximately 70% confluent and then pre-incubated with assay medium for several hours before treatments. Subsequently, the effective concentration of chrysin or 0.1% DMSO were added to the cells, and the cells were incubated at 37 °C in 5% (v/v) CO_2_ for the selected effective treatment time. Following chrysin treatment, samples were subjected to examination of cytoplasmic lipid accumulation, detection of apoptotic DNA fragments, TUNEL assay and extraction of total RNA, as described below.

### 3.5. Examination of Cytoplasmic Lipid Accumulation

Cytoplasmic lipid accumulation in chrysin-treated and control DMSO-treated MDA-MB-231 cells was examined using the Oil-Red O staining. The cells grown on culture slides were fixed with Fixation Solution (4% paraformaldehyde in PBS, pH 7.4) before being stained with Oil-Red O solution (Sigma-Aldrich) at 37 °C for 2 h. Samples were then counter-stained with Mayer hematoxylin solution (Sigma-Aldrich) and mounted with aqueous mounting medium. The morphology of the stained cells was examined using a Nikon eclipse TS100 inverted microscope at 200× magnification and the pictures were taken with a Nikon COLPIX digital camera.

### 3.6. Detection of Apoptotic DNA Fragments

Apoptotic DNA fragment detection in chrysin-treated and control DMSO-treated MDA-MB-231 cells was performed using the Calbiochem Suicide Track DNA Ladder Isolation Kit, according to the manufacturer’s instructions. For this purpose, samples grown in culture dishes were harvested and re-suspended in the lysis buffer provided in the kit. The DNA was extracted and subjected to electrophoresis on 1% agarose gels containing 0.1 μg/mL EtBr. Samples on the gel were visualized and photographed using the GeneSnap software on the Gene Genius Image Analyser.

### 3.7. TUNEL Assay

DNA fragmentation detection of TUNEL in chrysin-treated and control DMSO-treated MDA-MB-231 cells was performed using the Roche *In Situ* Cell Death Detection Kit, Fluorescein, according to the manufacturer’s instructions. For this purpose, samples grown on culture slides were dried and fixed with freshly prepared Fixation Solution, as described above. Cells were then incubated in a Permeabilisation Solution (0.1% Triton X-100 in 0.1% sodium citrate) before being re-suspended in the TUNEL reaction mixture provided in the kit. Samples were mounted with aqueous mounting medium and observed under a fluorescence microscope (Nikon ECLIPSE TE2000-U). DNase I recombinant was used as positive control for this assay.

### 3.8. Extraction of Total RNA and cDNA Synthesis

Extraction of total RNA from chrysin-treated and control DMSO-treated MDA-MB-231 cells was performed using the TRI Reagent (Molecular Research Center), according to the manufacturer’s instructions. The integrity of total RNA extracted was confirmed using agarose gel electrophoresis, whilst purity and yield were measured using an Eppendorf spectrophotometer. Subsequently, 1 μg of total RNA per sample was reverse transcribed to cDNA using the Fermentas RevertAid™ H Minus First Strand cDNA Synthesis Kit, according to the manufacturer’s instructions. The success of cDNA synthesis was validated with PCR amplification using human GAPDH housekeeping gene.

### 3.9. Quantitation of Gene Expression at mRNA Level

Following cDNA synthesis, levels of PPAR mRNAs in chrysin-treated and control MDA-MB-231 cells were semi-quantified using PCR and gel densitometry. The levels of mRNAs were expressed as the band intensity of PPAR PCR products relative to the control GAPDH PCR product. The student’s t-test was used to compare the expression of PPAR mRNAs between chrysin-treated and control DMSO-treated cells. The primers used for the PCR reactions are detailed in Table 2. PCR reactions were performed in a total volume of 25 μL consisting of 2.0 μL of cDNA, 0.3 μL of 10 mM dNTP Mix, 2.0 μL of 25 mM MgCl_2_, 2.0 μL of 10× *Taq* Buffer with (NH_4_)_2_SO_4_, 0.2 μL of *Taq* DNA Polymerase, 1.0 μL of each 20 pmol forward and reverse primers and DNase/RNase-free water. Each PCR reaction was carried out in triplicate and the reaction conditions were set as below for 35 cycles:

Pre-amplification: 94 °C for 2 min
Denaturation: 94 °C for 45 s
Annealing: 60 °C for 30 s
Elongation: 55 °C for 30 s
Extension: 72 °C for 1 min
Termination: 73 °C for 10 min

The PCR products were subjected to electrophoresis on a 0.8% (w/v) agarose gel containing 0.1 μg/mL EtBr. Bands of PCR product on the gel were visualized and photographed using the Gene Genius Image Analyser, and the band intensity was analysed using the LabImage 1D 2006 Professional software (KAPELAN Bio-Imaging Solution).

### 3.10. Statistical Analysis

All graph drawing and statistical calculations were performed using *GraphPad* software. The experiments were repeated several times to confirm the reproducibility of the results. All values are expressed as mean ± SD. A *p* value of less than 0.05 was regarded as statistically significant.

## 4. Conclusions

This study identified chrysin to be a potential inhibitor on the growth of MDA-MB-231 cell line. Our study, for the first time, provides mechanistic evidence that chrysin treatment inhibits the growth of MDA-MB-231 cells and results directly or indirectly in increased expression of PPARalpha mRNA. However, further studies are required to investigate the role of PPARalpha in the death mechanism of these cancer cells induced following chrysin treatment. Such studies would help to address the hypothesis for developing effective chrysin-based for human ER-negative breast cancer treatments.

## Figures and Tables

**Table 1.** The effects of chrysin in human and rat cell lines. Each study is denoted by the last name of the first author [5,7–10].

**Study**	**Cell type(s)**	**Concentration use**	**Title of study**
Parajuli *et al*. 2009	U87-MG, U251, MDA-MB-231 and PC3 cells	40–100 μM	*In vitro* anti-tumor mechanisms of various Scutellaria extracts and constituent flavonoids.
Izuta *et al*. 2008	SH-SY5Y cells	≤20 μM	Protective effects of Chinese propolis and its component, chrysin, against neuronal cell death *via* inhibition of mitochondrial-dependent apoptosis pathway in SH-SY5Y cells.
Weng *et al*. 2005	Rat C6 glioma cells	10–50 μM	Chrysin induces G1 phase cell cycle arrest in C6 glioma cells through inducing p21Waf1/Cip1 expression and involvement of p38 mitogen-activated protein kinase.
Woo *et al*. 2004	U937 promonocytic cells	≤10 μM	Chrysin-induced apoptosis is mediated through caspase activation and Akt inactivation in U937 leukemia cells.
Zhang *et al*. 2004	HeLa cells	≤30 μM	Chrysin and its phosphate ester inhibit cell proliferation and induce apoptosis in Hela cells.

**Table 2.** List of primers used for semi-quantitative RT-PCR. The primers were designed with an annealing temperature of 60 °C.

**Gene**	**Primer sequence**	**Product length**
PPARalpha	Forward: 5’-GCCAGTATTGTCGATTTCACAAGT-3’	344 bp
	Reverse: 5’-CTCCTTGTTCTGGATGCCATT-3’	
PPARdelta	Forward: 5’-CTGCTCACCAACAGATGAAGAC-3’	566 bp
	Reverse: 5’-ATAGCGTTGTGTGACATGCC-3’	
PPARgamma	Forward: 5’-CTTTATGGAGCCCAAGTTTGAG-3’	228 bp
	Reverse: 5’-GCTTCACATTCAGCAAACCTG-3’	
GAPDH	Forward: 5’-ACAGCCTCAAGATCATCAGCA-3’	137 bp
	Reverse: 5’-AGTCTTCTGGGTGGCAGTGAT-3’	
